# Notes

**Published:** 1885-04

**Authors:** 


To our readers we would say that any of them with time and means at their command can, by careful study of Dr. Johne's paper on Koch, and by experience and perseverance, perfect themselves in the methods of bacterial culture.

This paper is but the introduction of this subject to our readers; we have arranged not only with Prof. Johne, but with other eminent men in Europe, for occasional articles upon this and other important topics, and, as in the past, so in the future, shall spare no labor on our part to make this journal what we ourselves are determined it shall be—the best medical journal in the United States.

We would call the especial attention of our readers and exchanges to Dr. Bang's paper on tuberculosis in the udder of the cow, which we think is one of the most important articles we have ever published, and deserving the utmost attention of veterinarians, medical practitioners, Boards of Health and breeders. The few experiments here made should be repeated in large numbers, so that the evidence must be conclusive.

The duties of State Boards of Health and Agriculture are so plainly evident that comment would seem unnecessary.

Dairy inspection and the absolute isolation of every diseased and suspected cow, with the slaughter of the former, should be the inviolable law in every State.

---

Food Supply and Contagious Diseases.—It is generally admitted that tuberculosis and other contagious diseases may be communicated to man by means of diseased meat, milk, etc., and the guardians of the public health are becoming more and more convinced of the necessity of legislative action in the matter.

A bill has been lately introduced in the Senate and Assembly of the State of New Jersey, providing for the transmission of power from the State to the local Board of Health to appoint inspectors, subject to approval, who will give their whole attention to the detection of contagious animal diseases, impure milk, infected meat, etc.

It also provides for the punishment of those who deal in unwholesome food of whatever kind.

---

The careful reader of Dr. Biggs' article on cocaine will, we think, come to the conclusion that its physiological action certainly indicates the practical necessity of experimenting with it, subcutaneously, in cases of tetanus, for it seems as if it was especially indicated for this disease. We would also suggest its application in an experimental manner in the same way to what is called spasmodic colic. It should, certainly, at once give rest to the intestinal muscles.

---

Contagious Diseases on Long Island.—John Lindsay, D. V. S., of Huntington, L. I., has forwarded to us a statement of the contagious diseases which have come to his notice, as follows:

Anthrax made its appearance on October 14th, in a herd of twenty swine, with a mortality of 40 per cent.; on Oct. 27th, in a herd of fifty, with 10 per cent. mortality. Similar reports come from Babylon. C.

---

BACTERIA CULTIVATION.—The July number will contain an article from the pen of Dr. Billings on the above subject, with illustrations of the *modus operandi*, necessary instruments, &c.

---

HEAVES IN THE HORSE.—We are indebted to Dr. J. Gerth, Jr., of Newark, N. J., for the translation of the able article by Prof. Dickenhoff, entitled as above.

